# Circulating Cell-Free mtDNA Content as a Non-invasive Prognostic Biomarker in HCC Patients Receiving TACE and Traditional Chinese Medicine

**DOI:** 10.3389/fgene.2021.719451

**Published:** 2021-09-16

**Authors:** Guanlin Zhou, Ying Li, Shicheng Li, Hongxia Liu, Fei Xu, Xiaohuan Lai, Qiong Zhang, Jingxiang Xu, Shaogui Wan

**Affiliations:** ^1^Institute of Hepatology, Department of Hepatology, The Affiliated Fifth People’s Hospital of Ganzhou, Gannan Medical University, Ganzhou, China; ^2^Center for Molecular Pathology, Department of Basic Medicine, Gannan Medical University, Ganzhou, China; ^3^Department of Emergency Medicine, First Affiliated Hospital, Gannan Medical University, Ganzhou, China

**Keywords:** hepatocellular carcinoma, mitochondrial DNA, TACE, non-invasive biomarker, clinical outcome

## Abstract

Hepatocellular carcinoma (HCC) accounts for 70–85% of liver cancer, and about 85% of HCC are hepatitis B virus-related (HBV-HCC) in China. Transarterial chemoembolization (TACE) combined with traditional Chinese medicine (TCM) has been reported as an effective treatment. Potential biomarkers to stratify patients who may benefit from this treatment are needed. In this study, we aimed to evaluate whether circulating cell-free mitochondrial DNA (ccf-mtDNA) content was associated with the outcome of HCC patients, especially of those who received the combination treatment of TACE and TCM. Univariate and multivariate Cox analyses were conducted to evaluate the association between ccf-mtDNA content and the overall survival of HBV-HCC patients. Kaplan–Meier analysis was used to compare the survival differences between patients with low and high ccf-mtDNA content. In a hospital-based cohort with 141 HBV-HCC patients, there was no statistically significant association between the ccf-mtDNA content and the overall survival of HBV-HCC patients in the univariate analysis, but a borderline significant association was found in the multivariate analyses. In a subcohort of 50 HBV-HCC patients who received TACE and TCM treatment, high ccfDNA content conferred an increased death risk with a hazard ratio of 4.01 (95% confidence interval: 1.25–12.84, *p* = 0.019) in the multivariate analysis. Kaplan–Meier survival analysis also showed that patients with high ccf-mtDNA content had unfavorable survival (log rank *p* = 0.097). Our findings suggest that ccf-mtDNA content is a potential non-invasive prognostic biomarker in HCC patients receiving TACE and TCM treatment.

## Introduction

Hepatocellular carcinoma (HCC) is the second leading cause of cancer death, and chronic hepatitis B virus (HBV) infection accounts for at least 50% of HCC cases worldwide (Xie, 2017). Notably, HBV-related HCC accounts for about 85% of HCC cases in China. The vast majority of HCC patients are diagnosed at middle or later stages, and curative treatments like surgical resection are not suitable for these patients (Gomaa, 2015). Transarterial chemoembolization (TACE) is the standard of care for patients with intermediate HCC according to the Barcelona Clinic Liver Cancer staging system. Previous studies reported that TACE combined with traditional Chinese medicine (TCM) regimen displayed a high efficacy in treating advanced HCC (Chen et al., 2019).

The mitochondrion is a ubiquitous eukaryotic cell organelle which plays an important role in energy production, cell proliferation, as well as apoptosis (Veltri et al., 1990). The circular genome of the mitochondria (mtDNA) encodes for proteins essential in the oxidative phosphorylation system and the tRNA and rRNA molecules of the mitochondrial translation apparatus. Mitochondrial dysfunction plays an important role in the occurrence and development of liver cancer (Xiong et al., 2012; Dilip et al., 2013), and when mitochondrial dysfunction causes mtDNA damage, the mtDNA fragments can escape from the matrix and enter the cytosol or systemic circulation (Wenceslau et al., 2014). Changes in mtDNA content of tumor specimens have been reported recently (Reznik et al., 2016). The alteration of mtDNA content or sequence mutations has been involved in carcinogenesis and progression and thus becomes a potential predictive and prognostic biomarker for certain types of cancers (Fliss et al., 2000). Bao et al. (2016) found that the mtDNA copy number was associated with overall survival in HCC patients treated with TACE. mtDNA may also be released at low levels into the circulation from the mitochondria under cellular stress, which results in circulating cell-free mtDNA (ccf-mtDNA) being detectable in blood samples (Yu, 2012). Recently, circulating mtDNA was suggested to be a novel non-invasive biomarker for cancer diagnosis and prognostic evaluation due to its specific and unique characteristics (Li et al., 2016; Weerts et al., 2018), for example, the study of Li et al. (2016) showed that the serum ccf-mtDNA content of HBV-HCC patients was significantly lower than that of cancer-free HBV controls, and compared to HBV patients with high mtDNA content, those with low mtDNA content had a significantly increased risk of HCC. However, the association of ccf-mtDNA content with the clinical outcomes of HCC patients remains largely unknown.

In this study, using real-time quantitative PCR method, we measured the ccf-mtDNA content in a hospital-based cohort of HCC patients and further evaluated the association of ccf-mtDNA content with the clinical outcomes of HCC patients, especially of those who received TACE and TCM treatment.

## Materials and Methods

### Patients

**FIGURE 1:**
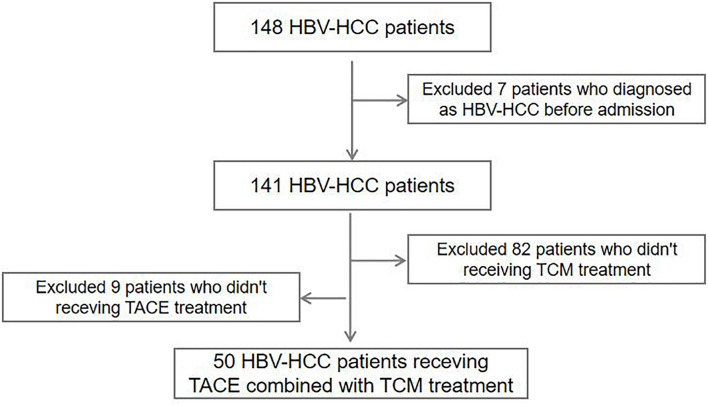
Flowchart of patient enrolment.

Informed consent was obtained from each patient according to the protocol approved by the ethics committee of the Affiliated Fifth People’s Hospital of Ganzhou in Gannan Medical University. A total of 148 HBV-HCC patients were enrolled at the Department of Hepatology, Affiliated Fifth People’s Hospital of Ganzhou in Gannan Medical University between January 2015 and October 2018. Each patient has two or more serum samples collected at different time points, and the first serum sample (baseline) after the patient was diagnosed with HCC was examined in this study. After excluding seven patients who had been diagnosed with HCC before their admission to this study, the final cohort included 141 patients with HBV-HCC (Figure 1). The statistical analysis was also conducted in a subcohort of 50 patients treated with TACE combined with TCM (Yangzhengxiaoji capsule, *n* = 23; Antike capsule, *n* = 5; and Huachansu capsule, *n* = 17; combination TCM treatments, *n* = 5).

### Serum Sample Preparation

Whole blood sample from each patient was collected in non-anticoagulated blood collection tubes and then transferred to the laboratory for processing within 6 h. The serum samples were collected by two-step centrifugation: the serum sample was extracted from the whole blood after centrifugation at 1,500 rpm for 5 min, then the supernatant was removed to a clean tube and re-centrifuged with 12,000 rpm for another 5 min, and finally, the supernatant was collected into a 1.5-ml tube and stored at −80°C for use.

### Measurement of Serum mtDNA Content

QIAamp DNA Blood Mini kit (Qiagen, Carlsbad, CA, United States) was used to isolate the circulating DNA from a 200-μl serum sample according to the protocol of the manufacturer. mtDNA content was measured by real-time qPCR with modified protocol as described by previous studies (Fu et al., 2012; Wan et al., 2012), in which the ratio of the copy number for mitochondrial ND1 gene to the copy of a human single copy gene 36B4 was used to determine the relative mtDNA content. Briefly, the primer of ND1 gene was used for mtDNA amplification with the primer sequences as follows: ND1-F: 5^μ^l qPCR reaction for ND1 and 36B4 consisted of 1× TB Green fast qPCR Mix (2×) (Takara), 0.2 μM of each primer, and 2 μl of purified serum DNA sample. The thermal cycling conditions were 95°C for 10 min, followed by 40 cycles of 95°C for 15 s, and 60°C for 60 s with signal acquisition. All samples were assayed in duplicate on a 96-well plate using the QuantStudio^TM^ 7 Flex qRT-PCR system (Applied Biosystems, United States). The same negative control and calibrator DNA samples were incorporated into each plate for quality control and the calibration of PCR efficiency. A reference DNA sample was used to construct a standard curve for mtDNA measurement in each plate. For each standard curve, the reference DNA sample was serially diluted by 1:3 to produce a six-point standard curve. The *R*^2^ for each standard curve was ≥0.99.

### Statistical Analysis

IBM SPSS 20.0 statistical software was used to analyze the collected clinical data and ccf-mtDNA. Continuous variables were represented by average with standard deviation (mean ± SD) or median with interquartile range, according to the data distributions. Categorical variables were described using frequency and percentage. Cox proportional hazards model was used for the univariate and multivariate analyses. Kaplan–Meier method was used to draw the survival curves, and log-rank test was used for comparisons of survival between groups. Moreover, *p*-value < 0.05 was considered as the threshold of statistical significance.

## Results

### Characteristics of HBV-HCC Patients

The epidemiological and clinical characteristics of 141 HBV-HCC patients are summarized in Table 1. The average age of the study population was 56.96 (SD 11.34) years, and most of the patients were male (83.7%), non-smokers (81.9%), non-drinkers (80.9%), and without a family history of cancer (73%). There were 45 (31.9%) patients who developed liver cirrhosis and 117 (83.0%) patients with a B or C Child–Pugh class. Among these 141 HBV-HCC patients, 70 (49.6%) patients were treated with TACE, 59 (41.8%) patients were treated with TCM, and 50 (35.4%) patients received a combination therapy of TACE and TCM. During a median follow-up of 28.0 months (interquartile range, 18.0–37.0 months), 88 (62.4%) patients have died.

**TABLE 1:** Characteristics of the study population.

**Characteristics**	**Number (*n* = 141, %)**
Age (median)	
≤57	71 (50.4)
>57	70 (49.6)
Gender	
Male	118 (83.7)
Female	23 (16.3)
Smoking	
No	114 (81.9)
Yes	27 (19.1)
Drinking	
No	114 (80.9)
Yes	27 (19.1)
Family history of cancer	
No	103 (73.0)
Yes	38 (27.0)
Liver cirrhosis	
No	96 (68.1)
Yes	45 (31.9)
Child-Pugh classification	
A	24 (17.0)
B	69 (48.9)
C	48 (34.1)
TACE treatment	
No	71 (50.4)
Yes	70 (49.6)
TCM adjuvant treatment	
No	82 (58.2)
Yes	59 (41.8)
Vital status	
Dead	88 (62.4)
Live	53 (37.6)
Follow-up time, month (median, interquartile range)	28.0 (18.0–37.0)
ccf-mtDNA content (median, interquartile range)	1.98 (1.17–3.68)

### Association Between ccf-mtDNA Content and Overall Survival of HBV-HCC Patients

The median and tertile values of ccf-mtDNA content were used to group patients, and the association between ccf-mtDNA content and the overall survival of HBV-HCC patients was evaluated using univariate and multivariate Cox analyses. As shown in Table 2, there was no statistically significant association between ccf-mtDNA content and the overall survival of HBV-HCC patients in the univariate analysis. However, in the multivariate analyses adjusting for age, gender, drinking and smoking status, history of family cancer, liver cirrhosis, Child–Pugh classification, and TACE treatment, a borderline significant association was observed. Compared to patients with low ccf-mtDNA, patients with high ccf-mtDNA content had an increased death risk with a hazard ratio (HR) of 1.49 (95%CI, 0.94–2.34, *p* = 0.089) when a median value of ccf-mtDNA was used as the cutoff or with a HR of 1.76 (95%CI, 1.00–3.11, *p* = 0.051, the highest vs. the lowest tertile) when tertile values were used as the cutoffs. If further adjusting for TCM adjuvant treatment, the association remained borderline significant in the tertile analysis (HR, 1.66; 95%CI, 0.93–2.94, *p* = 0.084).

**TABLE 2:** The association between ccf-mtDNA and overall survival of HBV-HCC patients.

**ccf-mtDNA**	**Number of patients**	**Number of deaths**	**Univariate**		**Multivariate^a^**		**Multivariate^b^**	
			**HR (95%CI)**	***P*-value**	**HR (95%CI)**	***P*-value**	**HR (95%CI)**	***P* value**
By median								
Lower	71	45	1.00		1.00		1.00	
Higher	70	43	1.07 (0.71–1.63)	0.740	1.49 (0.94–2.34)	0.089	1.44 (0.91–2.29)	0.120
By tertile								
1st tertile	47	28	1.00		1.00		1.00	
2nd tertile	47	32	1.45 (0.87–2.41)	0.156	1.58 (0.89–2.80)	0.122	1.60 (0.90–2.84)	0.111
3rd tertile	47	28	1.18 (0.70–1.99)	0.545	1.76 (1.00–3.11)	0.051	1.66 (0.93–2.94)	0.084
*P* for trend				0.364		0.127		0.156

*^a^Multivariate analysis adjusted for age, gender, drinking and smoking status, family history of cancer, liver cirrhosis, Child–Pugh classification, and TACE treatment.*

*^b^Multivariate analysis adjusted for age, gender, drinking and smoking status, family history of cancer, liver cirrhosis, Child–Pugh classification, TACE treatment, and TCM adjuvant treatment.*

**FIGURE 2:**
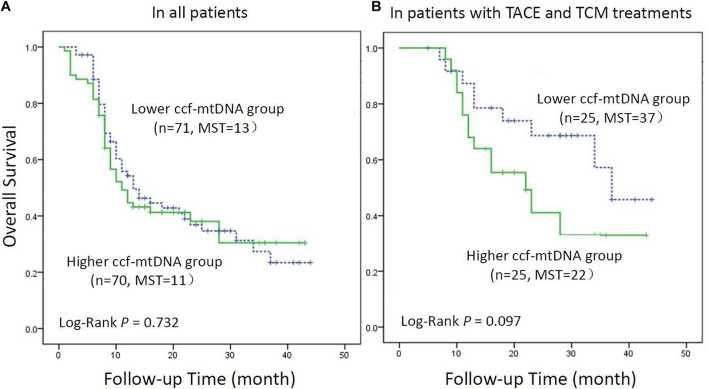
Kaplan–Meier curve analysis of ccf-mtDNA content and hepatitis B virus-related hepatocellular carcinoma (HBV-HCC) patients. **(A)** All HBV-HCC patients (*n* = 141). **(B)** HBV-HCC patients receiving transarterial chemoembolization combined with traditional Chinese medicine patients (*n* = 50). MST indicates the median survival time.

The Kaplan–Meier method was used to draw the survival curves of HBV-HCC patients with different ccf-mtDNA contents. As shown in Figure 2A, there was no statistically significant difference in the overall survival between patients with low and high ccf-mtDNA content (log rank *p* = 0.732).

### Association Between ccf-mtDNA Content and Overall Survival in HBV-HCC Patients Receiving TACE Combined With TCM

We further evaluated whether ccf-mtDNA content was associated with overall survival in a subcohort of patients receiving TACE combined with TCM. The epidemiological and clinical characteristics of 50 HBV-HCC patients in this subcohort are summarized in Table 3. The average age was 56.48 ± 8.18 years. Similar to the overall population, most of the patients were male (86%), non-smokers (70%), non-drinkers (70%), and without a family history of cancer (73%). There were 34 (68%) patients who developed liver cirrhosis and 36 (72%) patients with a B or C Child–Pugh class. Twenty-three (46%) patients died during a median follow-up of 29.0 months (interquartile range, 20.0–37.0 months).

**TABLE 3:** Characteristics of the HBV-HCC subcohort with TACE and TCM treatments.

**Characteristics**	**Number (*n* = 50, %)**
Age (median)	
≤55	25 (50.0)
>55	25 (50.0)
Gender	
Male	43 (86.0)
Female	7 (14.0)
Smoking status	
No	35 (70.0)
Yes	15 (30.0)
Drinking status	
No	35 (70.0)
Yes	15 (30.0)
Family history of cancer	
No	33 (66.0)
Yes	17 (34.0)
Liver cirrhosis	
No	34 (68.0)
Yes	16 (32.0)
Child–Pugh classification	
A	14 (28.0)
B	26 (52.0)
C	10 (20.0)
Tumor number	
Single	32 (64.0)
Multiple	18 (36.0)
Tumor size	
≤5	26 (52.0)
>5	24 (48.0)
Vital status	
Death	23 (46.0)
Live	27 (54.0)
Follow-up time (month) (median, interquartile range)	29.0 (20.0–37.0)
ccf-mtDNA content (median, interquartile range)	1.58 (0.77–3.27)

The patients were then stratified into different risk groups by the median or tertile values of ccf-mtDNA content. The results from multivariate analyses showed that the high content of ccf-mtDNA was an independent prognostic factor for HBV-HCC patients treated with both TACE and TCM (Table 4). A significantly higher death risk was observed in patients with high ccf-mtDNA content than in those with low ccf-mtDNA content (median cutoff: HR = 4.09, *p* = 0.018; tertile cutoff: HR 4.89, *p* = 0.021 in the middle tertile group, and HR 5.26, *p* = 0.039 in the highest tertile group) after adjusting for age, gender, smoking and drinking status, family history of cancer, liver cirrhosis, Child–Pugh classification, tumor number, and tumor size.

**TABLE 4:** The association between ccf-mtDNA and overall survival of HBV-HCC patients with TACE and TCM treatments.

**ccf-mtDNA**	**Number of patients**	**Number of deaths**	**Univariate**		**Multivariate^a^**	
			**HR (95%CI)**	***P*-value**	**HR (95%CI)**	***P*-value**
By median						
Lower	25	9	1.00		1.00	
Higher	25	14	2.02 (0.86−4.74)	0.097	4.09 (1.27−13.17)	0.018
By tertile						
1st tertile	17	6	1.00		1.00	
2nd tertile	17	10	2.66 (0.94−7.52)	0.066	4.89 (1.27−18.85)	0.021
3rd tertile	16	7	1.60 (0.53−4.85)	0.403	5.26 (1.09−25.31)	0.039
*P* for trend				0.173		0.038

*^*a*^Multivariate analysis adjusted for age, gender, drinking and smoking status, family history of cancer, liver cirrhosis, Child-Pugh classification, tumor number and tumor size.*

Kaplan–Meier analysis was used to assess the survival difference in HBV-HCC patients with different levels of ccf-mtDNA (Figure 2B). The result showed that the survival of patients with a high content of ccf-mtDNA was worse, but the difference in overall survival between the two groups did not reach statistical significance (log rank *p* = 0.097).

## Discussion

In this retrospective study, we measured the ccf-mtDNA content in HBV-HCC patients and demonstrated that patients with low ccf-mtDNA content had a survival benefit from TACE combined with TCM treatment. Moreover, the association between ccf-mtDNA and overall survival was independent of clinical confounders. A previous study showed that a low ccf-mtDNA content was associated with an increased risk for developing HBV-HCC (Li et al., 2016), while the current study suggested that the high level of ccf-mtDNA was associated with the poor outcome of HBV-HCC patients receiving TACE combined with TCM. The inconsistent findings may be explained by the different characteristics of the study populations, for example, ethnicity, tumor stage, liver function, and the treatments that the patients received.

ccf-mtDNA is the mtDNA fragments that are released outside the cell and into the circulation by cell necrosis and secretion. In the last decade, ccf-mtDNA has been demonstrated to be a potential non-invasive biomarker in different types of disease, including cancers. Several studies (Ellinger et al., 2012; Mahmoud et al., 2015; Li et al., 2016) showed that ccf-mtDNA in plasma or serum could be used as a diagnostic and prognostic marker in many solid tumors. The research of Ellinger showed that the ccf-mtDNA content in the serum of patients with urinary system malignancies increased significantly (Ellinger et al., 2012), and the study of Mahmoud showed that ccf-mtDNA had a prognostic value in breast cancer (Mahmoud et al., 2015). Meng et al. (2019) reported that increasing levels of ccf-mtDNA had a significant association with epithelial ovarian cancer progress and poor survival. These studies suggested that ccf-mtDNA is a potential tumor molecular marker, and our study further verified that ccf-mtDNA content in serum samples could be a potential non-invasive prognostic biomarker for HCC patients.

Transarterial chemoembolization is the first line of treatment for patients with intermediate stage of disease, including asymptomatic patients with limited unresectable multinodular lesions, without vascular invasion or extrahepatic spread, and who have well-preserved liver function (Raoul et al., 2019), although its clinical benefit is still far from satisfactory. A meta-analysis showed that TCM treatment improves the immune response for unresectable HCC after TACE treatment (Meng et al., 2011). TCM is gathering increasing interest due to the immunoregulatory properties of certain compounds which can restore immunosurveillance in HCC to promote anti-tumor effects in several pathways, including the upregulation of immunostimulatory factors and the downregulation of immunosuppressive factors (Jia and Wang, 2020). Several clinical studies have demonstrated that the utility of TCM can reduce adverse events after the TACE treatment of HCC patients. Tang et al. (2016) found that the Chinese medicine Jianpi Ligan decoction was effective in in reducing side effects and improving the long-term survival of patients with unresectable HCC treated by TACE. However, the cellular and molecular mechanisms of TCM-mediated anti-tumor effect are still not clear, and effective predictive and prognostic biomarkers are needed to increase the efficacy and improve the survival of HCC patients receiving treatment in the combination of TACE and TCM. In addition, mtDNA is also a well-known immune stimulator in many diseases including HCC. These previous findings may partially explain why, in this study, mtDNA content was associated with improved survival only in HCC patients who received both TACE and TCM, but not in the overall patient cohort. In our study, we also found that a high content of ccf-mtDNA was an independent prognostic factor for HBV-HCC patients receiving TACE combined with TCM treatment, suggesting its potential as a biomarker for identifying HCC patients who may benefit from this combination therapy.

There are several limitations in our study, for example, the small sample size (*n* = 50) in the subcohort may lead to an unstable estimation of the association between ccf-mtDNA content and clinical outcome of HBV-HCC patients receiving TACE combined with TCM treatment. In addition, the lack of pathological tumor stage in the population may not comprehensively address the confounding effects in the multivariate analysis. Therefore, future studies with a large sample size and comprehensive clinical parameters are needed to verify these findings.

In summary, this study showed that the ccf-mtDNA content in serum had a prognostic value for HBV-HCC patients receiving TACE combined with TCM treatment and may be used as a potential biomarker for the outcome prediction of HCC patients.

## Data Availability Statement

The raw data supporting the conclusions of this article will be made available by the authors, without undue reservation.

## Ethics Statement

The studies involving human participants were reviewed and approved by The Affiliated Fifth People’s Hospital of Ganzhou in Gannan Medical University. The patients/participants provided their written informed consent to participate in this study.

## Author Contributions

SW and GZ conceived and designed the experiment and analyzed the data. GZ and JX wrote the manuscript. YL, SL, and HL performed the experiments. FX, XL, and QZ collected the samples and clinical data. All authors are in agreement with the content of the manuscript and this submission.

## Conflict of Interest

The authors declare that the research was conducted in the absence of any commercial or financial relationships that could be construed as a potential conflict of interest.

## Publisher’s Note

All claims expressed in this article are solely those of the authors and do not necessarily represent those of their affiliated organizations, or those of the publisher, the editors and the reviewers. Any product that may be evaluated in this article, or claim that may be made by its manufacturer, is not guaranteed or endorsed by the publisher.
